# Vitamin D and Nonskeletal Complications among Egyptian Sickle Cell Disease Patients

**DOI:** 10.1155/2018/3867283

**Published:** 2018-09-16

**Authors:** Mona Hamdy, Niveen Salama, Ghada Maher, Amira Elrefaee

**Affiliations:** ^1^Department of Pediatrics, Faculty of Medicine, Cairo University, Cairo, Egypt; ^2^Department of Chemical Pathology, Faculty of Medicine, Cairo University, Cairo, Egypt

## Abstract

Lower levels of vitamin D have been documented in many patients with sickle cell disease (SCD), but data are still inconclusive regarding the association between vitamin D deficiency (VDD) and the occurrence or the severity of various SCD complications. Our study aimed to detect the prevalence of vitamin D deficiency among Egyptian patients with SCD and to associate it with the clinical course of the disease. We measured the level of 25-hydroxy vitamin D in 140 children (age from 4.3 to 15.5years), 80 patients with SCD and 60 controls using enzyme-linked immunosorbent assay. Vitamin D was deficient in 60% of SCD compared to 26.7% of controls. Severe VDD was significantly higher in SCD patients than controls. Patients were divided into 2 groups; Normal group (32 patients) and Deficient group (48 patients). There were statistically significant differences between the 2 groups regarding their age, height percentile, the presence of clinical jaundice, and osseous changes (P values 0.043, 0.024, 0.001, and 0.015, respectively). Hemoglobin and hematocrit values were significantly lower in Deficient group (P values 0.022 and 0.004, respectively) while the levels of aspartate aminotransferase, lactate dehydrogenase, and total and indirect bilirubin were significantly higher in the same group (P values 0.006, 0.001, 0.038, and 0.016, respectively). The frequency of blood transfusions, hospitalization, and vasoocclusive crisis previous year as well as the history of bone fracture and recurrent infections proved to be significantly higher in Deficient group. These findings suggest that VDD may play a role in the pathogenesis of hemolysis and other complication of SCD. Vitamin D monitoring and supplementation in patients with SCD should be implemented as a standard of care to potentially improve health outcomes in these affected patients.

## 1. Introduction

Vitamin D has been the focus of attention of many researchers concerned with general health as well as specific diseases. Though exposure of the skin to the ultraviolet (UV) sun rays is the main source of de novo vitamin D synthesis [1], North African and Middle Eastern countries, with abundant sun all over the year, reported the highest frequencies of vitamin D deficiency (VDD) in all age groups world-wide [2, 3].

Studies in patients with sickle cell disease (SCD) revealed high prevalence of VDD in these patients regardless of their age or ethnic background [4–7]. Predisposing factors that can contribute to such deficiency include decreased synthesis of vitamin D from sunlight due to skin pigmentation and limited outdoor activity, diminished exogenous supply as a result of poor appetite and impaired absorption by the damaged intestinal mucosa as a complication of SCD, and increased metabolic requirements due to increased erythrocyte production to compensate for shortened lifespan of the red cells and also decreased level of vitamin D binding protein ‘which is known in inflammatory conditions as SCD' resulting in decreased serum level of vitamin D. Finally impaired renal function which is known in many patients with SCD interferes with hydroxylation of vitamin D to 25-hydroxy-vitamin D (25-OHD) [4, 8].

Vitamin D deficiency has been linked to many skeletal and extraskeletal disorders including cardiovascular diseases [9], respiratory disorders, and asthma [10]. Vitamin D also has immunomodulatory and antimicrobial activities that affect both innate and acquired immunity [11]; all these disorders could have direct impact on the clinical course of SCD. In addition, suboptimal vitamin D levels have been detected in many pathological conditions associated with SCD such as vasoocclusive crises (VOC) [8], chronic pain [12], bone fragility [13], renal impairment [14], and autoimmune and inflammatory disorders [15]. Whether VDD initiates or exacerbates these disorders and the effect of its supplementation on their clinical courses remains to be determined.

Few studies are available regarding the nutritional status of SCD patients, including their vitamin D levels, and even fewer correlate specific nutritional deficiencies to the clinical profile of these patients.

*Aim of Work. *Our study was conducted to determine vitamin D status in Egyptian children and adolescents with sickle cell disease and to detect the effect of its deficiency on the clinical course of the disease as regards VOC, hemolysis, and other complications of the disease.

## 2. Materials and Methods

This is a case control cross-sectional study conducted on the Hematology Clinic, New Children Hospital, Cairo University. Eighty SCD patients and 60 age and sex frequency matched healthy control were enrolled. Informed consent was obtained from all patients, controls, and/or their legal guardians. Our study was conducted in accordance with the Declaration of Helsinki and was approved by the ethical committee of the New Children Hospital, Cairo University. All cases were in steady state indicated by the absence of any painful episodes in last 4 weeks prior to enrollment [17]. Patients older than 20 years and those with renal impairment, chronic malabsorption, osteoporosis, osteopenia, intercurrent infection or inflammation, and ongoing steroid therapy were excluded from the study. Clinical data were obtained from the patients' interviewing and medical records including anthropometric measures, number of blood transfusions, VOC, and hospital admissions in the last year.

VOC were classified as mild, moderate, and severe where mild and moderate VOC were managed at home (with non-steroidal anti-inflammatory drugs and weak opioid, respectively) while severe VOC required hospitalization and the use of strong opioid [18].

The cross-sectional nature of the study and the limited financial resources due to self-funding of the study hinder our ability to monitor vitamin D level all over the year, so blood sampling was performed during the summer months ‘from June to August', taking into consideration the seasonal variation of vitamin D level with expected higher level of the vitamin during summer, assuming that deficient patients during summer are deficient in the other seasons.

Blood was withdrawn from all study populations in the outpatient clinic. Laboratory investigations included complete blood count, reticulocyte count, aspartate transaminase (AST), alanine transaminase (ALT), lactate dehydrogenase (LDH), and total and indirect serum bilirubin.

### 2.1. 25-Hyroxyvitamin D

25-OHD was measured for case and control groups as it is the major circulating form of vitamin D and is considered the most accurate marker for vitamin D status [19].

### 2.2. Sample Collection, Storage, and Preparation

Blood samples were collected by venipuncture, allowed to clot, and then centrifuged at room temperature for 1 hour. Specimens were stored at 8 Celsius degrees for 3 days and or -20 Celsius longer duration.

### 2.3. Assay Procedures

Enzyme-linked immunosorbent assay (ELISA) was based on competitive binding using the DRY-HYBRID-XL 25-OH Vitamin D kits. The walls of the reagents cartridge are coated with vitamin D binding protein (VDBP). Endogenous 25-OHD of the samples competes with a 25-OHD-biotin for binding to the coated VDBP. After incubation the unbound conjugate is washed off, thereafter, bound 25-OHD-biotin conjugate is detected by streptavidin-conjugated peroxidase (Enzyme complex). The amount of bound peroxidase conjugate is inversely proportional to the concentration of 25-OHD in the sample [20].

### 2.4. Expected Normal Values

Each laboratory should determine its own normal and pathological values. Our laboratory suggests the ranges in Table 1 for the classification of 25-OHD status [16].

The dynamic range of the assay is defined by the limit of detection and the maximum values of the Master curves. Values found below the measuring range are indicated as < 4.6 ng/mL and values above the measuring ranges are indicated as > 130 ng/ml.

### 2.5. Statistical Analysis

The data were analyzed using the S-plus Statistics Software* (SPSS version 21)*. Descriptive statistical calculations (mean ± standard deviation) were done to quantitative values. Statistical analyses were performed using the independent* t*-test as applicable for quantitative variables.* Fisher exact test *and* Pearson Chi-Square test* were used for qualitative variables.* Two-tailed P-values* of less than 0.05 were considered to be significant.* The Pearson correlation coefficient* (r) was used to express the relationship between quantitative variables in different groups.

## 3. Results

The study populations were composed of 80 Egyptian cases with SCD, compared to 60 age and sex frequency matched healthy controls with male-to-female ratios 1.4: 1 and 1.3:1, respectively. Case group consisted of 59 (73.8%) patients with homozygous hemoglobin S* (HBSS)* and 21(26.2%) patients with sickle *β*-thalassemia* (HBSβ)*. The demographic and laboratory data of the 2 groups are shown in Table 2.

There were statistically significant differences between case and control groups regarding the level of hemoglobin (Hb), hematocrit, mean corpuscular volume (MCV), mean corpuscular hemoglobin (MCH), and white blood cell (WBC) counts.

Cases of sickle cell disease were divided into 2 groups: ‘Normal group' with normal vitamin D level (32 patients) and ‘Deficient group' with VDD (48 patients).  Table 3 showed the demographic and laboratory data of the 2 groups.

There were statistically significant differences between the 2 groups regarding their age, height percentile, and the presence of clinical jaundice and osseous changes (P values 0.043, 0.024, 0.001 and 0.015, respectively). Regarding lab results, hemoglobin and hematocrit values were significantly lower in Deficient group (P values 0.022 and 0.004, respectively) while the levels of aspartate aminotransferase, lactate dehydrogenase, and total and indirect bilirubin were significantly higher in the same group (P values 0.006, 0.001, 0.038, and 0.016, respectively).

Data from Table 4 shows weak but statistically significant correlation between serum 25-OHD and the biomarker of hemolysis and red blood cell turnover. VDD patients had lower level of hemoglobin and hematocrit and higher level of AST, LDH, and total and indirect bilirubin.

The association between VDD and the clinical course and complications of SCD is shown in Table 5.

The frequencies of blood transfusions and hospitalization last year were significantly higher in Deficient group; also the occurrence of vasoocclusive crises last year and the presence of old fractured bone and recurrent infections proved to be statistically significant.

## 4. Discussion

The reported incidences of VDD range from 20 to 80% in some Middle Eastern counties [21]. Despite these high incidences, VDD often remained underdiagnosed and untreated especially in patients with SCD where chronic pain of VDD was usually credited to SCD, as the pain in both conditions is dull aching and deeply seated and involves the back and extremities [22].

In our study, we found 26.7% incidence of VDD among the healthy control group; however, further population based studies are needed to evaluate vitamin D status among the Egyptians. Forty-eight (60%) of our SCD patients and 16 (26.7%) of controls were found to have VDD, with 13 cases and 5 controls having severe deficiency with statistically significant difference between the two groups. These results are in close approximation to those obtained from Spain [23] and Turkey [24] where the prevalence of VDD among SCD patients was 56.4% and 63.1%, respectively, while the prevalence of severe VDD ( 25-OH D level < 10 ng/ml) was 12% in a study on sickle patients conducted in Saudi Arabia [25].

We detected a weak negative correlation between age and vitamin D level; the older the age, the lower the level. This correlation ‘which is well established by other studies [1, 26]' could be weak due to the proximity in age distribution between our studied patients with VDD and those with normal vitamin D level. In older age patients, this negative correlation could be explained by prolonged course of the disease with more skin pigmentation, more intestinal mucosal damage, and renal impairment which all affect vitamin D metabolism.

We did not detect any significant correlation between sickle disease genotype and vitamin D status. This finding was supported by similar study performed on SCD patients in Spanish population [23].

In our study, we found significantly lower height percentile in SCD patient with VDD than those with normal vitamin D level, while the weight percentile was lower though did not reach significance. Other studies [24, 25] showed impairment in both weight and height percentile among SCD patients with VDD compared to those with sufficient vitamin D. Further verification studies are needed to detect the impacts of VDD on the patients' growth parameters.

A statistically significant correlation was observed between vitamin D level and biomarkers of hemolysis. Lower vitamin D levels were associated with lower hemoglobin and hematocrit but with higher reticulocyte counts, AST, LDH, and total and indirect serum bilirubin levels. Whether VDD increases hemolysis or excessive hemolysis increases the demand of vitamin D is still debatable. Winters and colleagues suggested that increased hemolysis and bone marrow activity may interfere with vitamin D absorption leading to VDD [26], while other authors suggested that VDD may increase hemolysis of RBCs in patient with SCD [19].

Low “hemoglobin and hematocrit” and high “reticulocyte count and LDH” were associated with significantly lower level of vitamin D in other studies [27–29]. A recent study showed a positive correlation between hemoglobin concentration and vitamin D level where 1g/L hemoglobin increase was associated with 0.4 nmol/L increase of serum vitamin D level [30]; however, others showed nonsignificant correlation between vitamin D level and hemoglobin, hematocrit, reticulocyte count, or AST level [5, 19, 31].

We have noticed that, throughout the year prior to enrollment, SCD patients with VDD had significantly higher incidences of VOC, blood transfusions, hospital or emergency room visits, and recurrent infections as compared to those with normal vitamin D level.

Vasoocclusive crisis is the hallmark of SCD and the main cause of healthcare utilization. Our study along with other studies [8, 32] provided evidence that painful episodes in SCD correlate positively with VDD, the mechanism of which is still unclear, but a recent study associates lower vitamin D level to increased expression of* SLC6A5 gene* which encodes for a neuronal pain pathway protein called glycine tranporter-2 which may have a direct effect on the nervous system. Impaired bone health may also contribute to these painful episodes [32, 33]; Osunkwo and colleagues also proved that proper vitamin D therapy could reduce the number of painful days and improve quality of life [12]. Though others failed to detect any association between VDD and the number of painful episode, this could be explained by the high incidence (96.4%) of VDD among the studied sample [34].

The role of vitamin D in both innate and acquired immunity has been well established; vitamin D activated at extrarenal sites supports innate immunity by stimulating the expression of Cathelicidin, “member of a group of antimicrobial peptide called Defensins”, which is usually suppressed by pathogens [11]. Vitamin D also exhibits a pivotal role in both cell mediated and humoral immune responses by modulating the proliferation of T lymphocytes and regulating cytokines production and also through downregulation of B lymphocyte proliferation, antibodies production, and cell switching to plasma or memory cell [35, 36]. In our study, we detected a significantly higher incidence of respiratory and urinary tract infections among patients with VDD. Several studies conducted in children confirmed our findings of significant association between VDD and increased risk of viral and bacterial respiratory tract infections [37–39]; Urashima and colleagues even suggest that vitamin D supplementation plays a role in prevention of seasonal influenza [40]. Other authors encounter more than 50% reduction of the respiratory illness rate on the second year of monthly vitamin D supplementation [41].

Our study has its own limitations: first, the studied sample being collected from patient attending SCD clinic at a single tertiary care hospital which may not represent all patients with SCD; second, the lack of community-based studies to detect the prevalence of VDD among the Egyptian population; lastly, the lack of proper dietary and medication history including vitamins supplementation.

## 5. Conclusions and Recommendations

Vitamin D deficiency is a major nutritional health problem in patients with sickle cell disease that may aggravate the disease process and increase the risk of its complications. Further prospective and interventional studies are needed to confirm the causal relationship between VDD and suspected complications and the effect and proper dose of vitamin D replacement before considering vitamin D as an adjunct therapy in SCD management; however, periodic measurement of vitamin D level should be implemented as primary care point in patients with SCD.

## Figures and Tables

**Table 1:** The ranges for the classification of 25-hydrxyvitamin D status [16].

**Vitamin D status**	**25-OH Vitamin D (ng/ml)**
Severe deficiency	<10

Deficiency	10-20

Sufficiency	20-30.

Toxicity	>100

**Table 2:** Demographic and laboratory data of the case and control groups.

**Variable**	**Case(n=80)**	**Control(n=60)**	***P*-value**
Age: in years:median (IQR)	9 (7.5)	9.5 (8)	0.753

Sex: n (%)			0.522
Male (%)	47 (58.8%)	34 (56.6%)	
Female (%)	33 (41.2%)	26 (43.3%)	

Hemoglobin (g/dl): mean ± SD	8.3 ±1.4	13.2 ±1.8	**Less than 0.001**

Hematocrit (%): mean ± SD	25.5 ±4.8	37 ±3.9	**Less than 0.001**

MCV (FL): mean ± SD:	84.5 ±8.8	88.1 ± 6.8	**0.008**

MCH (pg): mean ± SD	25.0 ±3	32.3 ±1.7	**Less than 0.001**

WBC (10^3^mm^2^): mean ± SD	9.2 ±4.4	7.9 ±2.1	**0.033**

Platelets (10 ^3^mm^2^): mean ± SD	328.2 ±164.4	284.7 ±106.4	0.067

25-OHD (ng/ml): mean ± SD	22 ±10.4	22.7±8.4	0.658

No Deficiency: n (%)	32 (40%)	44(73.3%)	0.061

Deficiency: n (%)	48 (60%)	16 (26.7%)	0.058

Severe deficiency: n (%)	13 (16.2%)	5 (8.3%)	**0.021**

25-OHD in VDD groups (ng/ml): mean ± SD	9.9 ±2.1	11±1.8	**0.049**

n= number. IQR= interquartile range. SD = standard deviation. 25-OHD =25-hydroxyvitamin D. MCV = mean corpuscular volume. MCH= mean corpuscular hemoglobin. WBC= white blood cells. VDD= vitamin D deficiency. Bold values indicate statistical significance.

**Table 3:** Demographic and laboratory data of the 2 groups.

**Variable**	**Normal Group (n=32)**	**Deficient Group (n=48)**	***P*-value**
Age: in years:median (IQR)	8 (9.235)	11(5)	**0.043**

Sex: n (%)			0.732
Male (%)	17 (53.1%)	30 (62.5%)	
Female (%)	15 (46.9%)	18 (37.5%)	

Weight percentile: mean ± SD	29.7±20.2	22.1±18.2	0.072

Height percentile: mean ± SD	33.8±26.5	22.2±24.8	**0.024**

Hemoglobin genotype: n (%)			0.635
*HBSS* (%)	26 (81.3%)	33 (68.7%)	
*HBSB+* (%)	5 (15.6%)	11 (20.8%)	
*HBSB0 *(%)	1 (3.1%)	4 (10.5%)	

Splenomegaly: n (%) (n= 28)	12 (37.5)	16 (33.4)	0.655

Splenectomy: n (%) (n=22)	7 (31.8)	15 (68.2)	0.156

Pallor: n (%) (n=38)	12 (31.5)	26 (68.5)	0.267

Jaundice: n (%) (n=21)	1 (4.8)	20 (95.2)	**0.001**

Osseous changes: n (%) (n=13)	3 (23)	10 (76)	**0.015**

Hemoglobin (g/dL): mean ± SD	8.6±1.1	7.9±1.6	**0.022**

Hematocrit (%): mean ± SD	26.9±4	23.9±5.1	**0.004**

Reticulocyte count (%): mean ± SD	4.8±2.9	6.5±4.1	0.063

Corrected Reticulocyte count (%): mean ± SD	2.6±1.4	3±2.1	0.401

AST (IU/L): mean ± SD	43.3±23.3	54.4±20.1	**0.006**

ALT (IU/L): mean ± SD	25.1±20.3	27.1±18.8	0.540

LDH (IU/L): mean ± SD	454.8±279.4	634.9±277.8	**0.001**

TSB (mg/dL): mean ± SD	4.2±2.4	5.9±3.5	**0.038**

Indirect bilirubin (mg/dL): mean ± SD	2±1.8	2.6±1.4	**0.016**

n = number. IQR = interquartile range. SD = standard deviation. * HBSS*= homozygous hemoglobin S; *HBSβ*^*+ *^= S*β*^+^ thalassemia; *HBSβ*^*0*^ = S*β*^0^ thalassemia; AST = aspartate aminotransferase; ALT = alanine aminotransferase; LDH = lactate dehydrogenase; TSB = total serum bilirubin. Bold values indicate statistical significance.

**Table 4:** Correlation between Serum 25-OHD and biomarkers of intravascular femolysis.

**Variable**	**Correlation Coefficient(r)**	***P.*value**
Hemoglobin (g/dl)	0.27	**0.019**
Hematocrit (%)	0.29	**0.011**
Reticulocyte count (%)	-0.31	**0.007**
Corrected Reticulocyte count (%)	-0.22	0.061
AST	-0.33	**0.003**
ALT	-0.05	0.618
LDH	-0.27	**0.017**
TSB	-0.29	**0.010**
Indirect SB	-0.35	**0.002**

AST = aspartate transaminase; ALT = alanine transaminase; LDH = lactate dehydrogenase; TSB = total serum bilirubin; indirect SB = indirect serum bilirubin. Bold values indicate statistical significance.

**Table 5:** Association between VDD, clinical course, and complications of SCD.

**Variable**	**Normal Group (n=32)**	**Deficient Group (n=48)**	***P*-value**
Blood transfusion: n (%) (n=69)	30 (43.4)	39 (56.6)	0.475

Age at 1^st^ blood transfusion (years)	2.3 ± 1.9	1.6 ±1.0	0.351

Frequency of blood transfusion/ Last year	3.6 ± 2.8	5.4 ± 3.5	**0.021**

Positive VOC/ Last year: n (%) (n=68)	24 (35.2)	44 (64.8)	**0.032**

Degree of VOC: n (%)			0.732
Mild (n=41)	13 (31.7)	28 (68.3)	
Moderate (n= 21)	10 (47.6)	11 (52.4)	
Severe (n= 6)	1 (16.6)	5 (83.4)	

Hospital admission/ Last year: n (%) (n=44)	14 (31.8)	30 (68.2)	**0.012**

Bone fracture: n (%) (n=8)	1 (12.5)	7 (87.5)	**0.034**

Recurrent infection: n (%) (n= 36)	7 (19.5)	29 (80.5)	**0.036**

Viral hepatitis (B&C): n (%) (n=12)	5 (41.6)	7 (58.4)	0.219

Pulmonary hypertension: n (%)(n=11)	3 (27.3)	8 (72.7)	0.068

Diabetes Mellitus: n (%) (n=4)	2 (50)	2 (50)	0.721

Abnormal TCD: n (%) (n= 2)	1 (50)	1 (50)	0.834

Gall Bladder stones: n ( %) (n= 3)	1 (33.3)	2 (66.7)	0.059

n = number; SD = standard deviation; VOC = vasoocclusive crisis; TCD= transcranial Doppler. Bold values indicate statistical significance.

## Data Availability

The data used to support the findings of this study are available from the corresponding author upon request.
